# Consideration of nutrition and sustainability in public definitions of ‘healthy’ food: an analysis of submissions to the US FDA

**DOI:** 10.1017/S1368980024000636

**Published:** 2024-04-04

**Authors:** Emily H Belarmino, Michelle Carfagno, Lauren Kam, Kene-Chukwu Ifeagwu, Miriam E Nelson, Rebecca A Seguin-Fowler

**Affiliations:** 1 Department of Nutrition and Food Sciences, University of Vermont, 109 Carrigan Drive, Burlington, VT 05405, USA; 2 Gund Institute for Environment, University of Vermont, 210 Colchester Ave, Burlington, VT 05405, USA; 3 Division of Nutritional Sciences, Cornell University, Savage Hall, Ithaca, NY 14853, USA; 4 Gerald J. and Dorothy R. Friedman School of Nutrition Science and Policy, Tufts University, 150 Harrison Avenue, Boston, MA 02111, USA; 5 Institute for Advancing Health through Agriculture, Texas A&M AgriLife Research, 1500 Research Parkway, Centeq Building B, College Station, TX 77845, USA

**Keywords:** Health, Nutrition, Sustainability, Food policy, Federal rulemaking

## Abstract

**Objective:**

To better understand how the public defines ‘healthy’ foods and to determine whether the public considers sustainability, implicitly and explicitly, in the context of healthy eating.

**Design:**

We conducted a content analysis of public comments submitted to the US FDA in 2016 and 2017 in response to an invitation for feedback on use of the term ‘healthy’ on food labels. The analysis explored the ways in which commenters’ definitions of ‘healthy’ aligned with the 2015–2020 Dietary Guidelines for Americans and whether their definitions considered sustainability.

**Setting:**

The US Government’s Regulations.gov website.

**Participants:**

All 1125 unique comments from individuals and organisations.

**Results:**

Commenters’ definitions of ‘healthy’ generally mirrored the recommendations that the Dietary Guidelines for Americans put forth to promote a ‘healthy eating pattern’. Commenters emphasised the healthfulness of fruit, vegetables, whole grains, fish and other minimally processed foods and the need to limit added sugars, sodium, saturated and *trans* fats and other ingredients sometimes added during processing. One-third of comments (*n* 374) incorporated at least one dimension of sustainability, mainly the environmental dimension. Commenters who mentioned environmental considerations primarily expressed concerns about synthetic chemicals and genetic modification. Less than 20 % of comments discussed social or economic dimensions of sustainability, and less than 3 % of comments (*n* 30) used the word ‘sustainability’ explicitly.

**Conclusions:**

This novel analysis provides new information about the public’s perceptions of ‘healthy’ foods relative to nutrition and sustainability considerations. The findings can be used to advance policy discussions regarding nutrition labelling and guidance.

Historically, federal dietary guidance and recommendations have focused on the promotion of nutritionally adequate diets and healthy lifestyles^(1)^. However, the consideration of food system sustainability as a component of nutrition policies has been proposed^(2)^ in recognition of the complex ways in which sustainability challenges may threaten nutrition security – or the ability of all members of the population to have ‘consistent and equitable access to healthy, safe, affordable foods essential to optimal health and well-being’^(3)^. Modern food systems are critical to meeting population food and nutrition needs, but also stress the natural resources upon which human nutrition and health depend. They are key consumers of land^(4–7)^, water^(5,6)^ and raw materials^(5)^; can contribute positively or negatively to air quality^(4,8)^, water quality^(4,9)^ and biodiversity^(7,10)^ and employ millions across diverse sectors including agriculture, processing, manufacturing and food service^(11)^. Beyond nutrition and health, the literature on food system sustainability typically considers three dimensions: environmental sustainability (the protection of natural resources), social sustainability (the protection of human resources and pursuit of social equity) and economic sustainability (the generation of human prosperity)^(12–14)^. Perturbations in any or all these dimensions have the potential to compromise human nutrition and health by reducing agricultural output^(15)^, increasing food contamination^(16)^, disrupting food supply chains^(7)^, reducing food quality^(17,18)^, increasing food prices^(7)^ and limiting food choices^(19)^.

Dietary guidelines are one policy tool through which health considerations and food system sustainability goals have an opportunity to align. The US Department of Health and Human Services and the US Department of Agriculture update and publish the Dietary Guidelines for Americans (DGA) every 5 years, informed by a review of the research by the Dietary Guidelines Advisory Committee (DGAC), an expert scientific panel. Based on its review, the DGAC recommended, for the first time in 2015, that food system sustainability be incorporated into the DGA^(20)^. This suggestion generated considerable public engagement; more than 29 000 comments were submitted to the federal government about the DGAC report, about half of which addressed the issue of including sustainability in the DGA^(21,22)^. Following review of these comments, the two US government Cabinet Secretaries that oversee the writing of the DGA released a statement that sustainability was beyond the scope of the mandate for the DGA and ultimately opted not to include sustainability language in the 2015–2020 DGA^(23)^. The 2020 and 2025 DGAC were not charged with updating the review of research on links between dietary patterns and sustainability. Although there remains debate about how synergies across food-related policies, programmes and guidelines can be achieved^(24,25)^, some countries such as Brazil, Germany, Qatar and Sweden have expanded the scope of their dietary guidance in recent years to incorporate aspects of sustainability following stakeholder input and consultation on how to effectively encourage to better food choices^(12,14,26)^. For example, the Brazilian guidelines discuss the sustainability impacts of different dietary patterns and provide health, environmental, social and economic rationale for their recommendations^(26)^.

Food labels, primarily found on packaged foods, represent another policy instrument that can potentially help address challenges related to both human health and food system sustainability. Packaged foods tend to be higher in sodium, added sugars and refined grains, and they often carry the burden of sustainability impacts as well^(27–30)^. For example, highly processed, packaged foods have been associated with intensive resource use^(30)^, greenhouse gas emissions^(30)^, biodiversity loss^(30)^ and food and plastic waste^(30)^ and some there are some concerns that their supply chains may redirect food spending away from small producers^(31)^. Health- and nutrition-related claims are widely used on packaged food labels; yet research suggests some may be misleading^(32,33)^, and some advocates have called for changes to labelling regulation^(34,35)^. In the USA, the present regulatory definition allows a packaged food to bear a ‘healthy’ nutrient content claim if it is low in fat and low in saturated fat as defined by the US FDA, meets certain criteria for cholesterol and sodium content, and serves as a good source of vitamin A, vitamin C, calcium, iron, protein or fibre^(36)^.

This formal definition of ‘healthy’ was formulated 30 years ago when nutrition science and policy focused on limiting fat intake. In September 2016, in an effort to increase policy coherence and respond to a citizen petition requesting changes to the regulation on the use of ‘healthy’ on labelling, the FDA issued a request for information and public comments on use of the term ‘healthy’ to describe foods, especially in the context of food labelling, and whether the term ‘healthy’ may be false or misleading (FDA-2016-D-2335)^(37)^. A broad set of questions was posed including ‘Are there current dietary recommendations (e.g. the Dietary Guidelines for Americans) or nutrient intake requirements… that should be reflected in criteria for use of the term “healthy?” What is consumers’ understanding of the meaning of the term “healthy” as it relates to food? What are consumers’ expectations of foods that carry a “healthy” claim?’ The deadline initially was set to January 2017 but was later extended to April 2017. Federal agencies must publish notices of proposed rulemaking in the Federal Register and provide the opportunity for any person or organisation to share insights and information in a comment before final rules can be put into effect. Agencies are required to consider public comments prior to publishing the final rule^(38)^. The FDA has proposed new labelling guidance but has not published its final rule on this issue. Under the new proposed definition, manufacturers can label their products ‘healthy’ only if they contain a meaningful amount of food from at least one of the recommended food groups or subgroups as outlined in the DGA and adhere to specific limits for saturated fats, sodium and added sugars^(39)^. Currently, and within the proposed definition as of August 2023, the ‘healthy’ label regulation does not include any sustainability dimensions.

Using data collected as part of the FDA solicitation in 2016 and 2017, we examined commenters’ definitions of ‘healthy.’ The aim of the research presented herein was to examine how commenters defined healthy with respect to the DGA and elucidate if there were implied or explicit mentions of the dimensions of sustainability.

## Methods

### Data

**Fig. 1:**
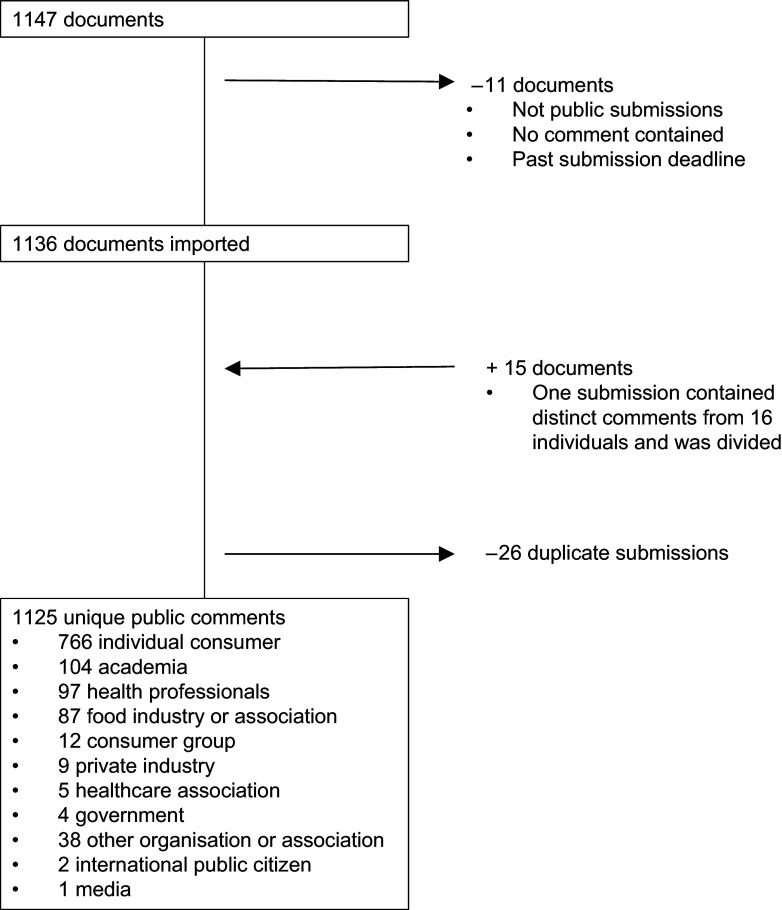
Flow diagram of comments submitted to FDA-2016-D-2335

Submissions to the Federal Register are publicly available at Regulations.gov^(37)^. We downloaded each comment submitted during the comment period (September 2016 – April 2017) and created a database that included the submitter’s name, location and category (e.g. individual consumer, food industry and academia). A total of 1136 public comments were submitted by the final deadline (Fig. 1). One submission was composed of sixteen distinct comments and was therefore divided. About 2 % (*n* 26) were determined to be duplicates (i.e. identical comments submitted > 1 time by the same person) and excluded. The final sample included 1125 unique comments. All data were imported into the NVivo qualitative data analysis software (QSR International Pty Ltd. Version 11) for coding.

### Analysis

A three-member team trained by the first author coded the data in a two-step process. First, to index and explore the data with respect to nutrition, we coded all comments for alignment with recommendations from the 2015–2020 DGA by identifying each reference to the main food groups (or foods in those groups) as well as sodium, added sugars, saturated fats and *trans* fats. We organised these codes into dietary factors that are ‘included’ or ‘limited’ in a healthy eating pattern as defined by the DGA^(40)^. Included dietary factors are vegetables, fruits, grains, low-fat and fat-free dairy, protein foods and oils, while sodium, added sugars, saturated fats and *trans* fats are defined as dietary factors to limit. To simplify our coding scheme, we considered all dairy products together (regardless of whether they were fat-free, low fat or had a higher fat content) and considered plant-based proteins (nuts, seeds and soya products) separate from animal-source protein foods (meat, fish and eggs). Additionally, we coded for references to food processing and serving sizes, as sodium, added sugars and saturated fats are often found in highly processed foods and eating within appropriate energy levels is recommended in the DGA^(40)^. Relevant codes are presented in Supplementary File 1.

Next, the first author developed a structural coding framework relevant to the three non-health dimensions of sustainability typically addressed in the literature on food systems: environmental, social and economic sustainability^(12–14)^. Sub-codes were created based on how the environmental, social and economic dimensions are defined in the United Nation’s Sustainability Assessment of Food and Agriculture Systems Guidelines^(41)^ and ideas that emerged in the first stage of coding. In line with Béné et al.^(13)^, we considered issues related to governance and power dynamics as part of social sustainability. The members of the coding team piloted the framework with twenty-five randomly selected comments and met to review coding decisions, discuss discrepancies and revise the codebook. To assess and ensure consistency before applying the codebook to the full dataset, we applied the updated codebook to another twenty-five randomly selected comments and compared coding decisions. The aspects of the final coding framework relevant to this analysis are presented in Supplementary File 1.

One member of the coding team coded each comment with the final codebook, and a second member of the team reviewed the coding decisions. For each code, we resolved disagreements by discussion. We analysed the data by reviewing each code and co-produced a corresponding summary report with information on themes and ideas in the data. We used matrix coding queries to compare comments between those who identified themselves as individual consumers and those who identified as another category of respondent. To complement the qualitative analysis, we generated code frequency reports based on the number of comments that included information related to each dimension of sustainability. To identify explicit mentions of sustainability, we conducted word searches for the terms ‘sustainable,’ ‘sustainably’ and ‘sustainability.’ Quotes from the comments are presented verbatim.

## Results

Two-thirds of comments were submitted by individual consumers (Fig. 1). The next most common types of commenters were those from academia, health professions or the food industry. As with individual consumers, most academic and health professional commenters were responding as private citizens. A substantial minority of those from academia were students submitting position papers. Responses were received from across the country and two came from individuals who specified a location outside of the US. Although 62·0 % of commenters did not report their location, of those based in the USA that did report (*n* 425): 18·1 % came from the Midwest Census division, 8·7 % from the Northeast Census division, 32·5 % from the South Census division and 26·4 % from the West Census division (data not shown).

### Alignment between comments and the recommendations in the 2015–2020 DGA

Submissions generally aligned with the key recommendations for foods and ingredients to include and limit in a healthy dietary pattern as defined in the 2015–2020 DGA (Table 1). Commenters identified vegetables, fruits, whole grains, nuts, seeds, legumes and naturally occurring oils (especially those present in plant foods, fish and seafood) as central to a healthy diet. Comments on meat tended to highlight either the perceived benefits of consuming minimally processed meats, lean meats, poultry, fish and seafood or the perceived risks associated with intake of red and processed meats. Comments on dairy revealed diverse views, particularly with respect to beneficial levels of fat (e.g. full fat *v*. low-fat or fat-free) and processing. A small number of commenters did not view any animal-source foods as part of a healthy diet.

**Table 1:** Perspectives on foods included and limited in a healthy dietary pattern as defined in the 2015–2020 DGA

Foods or ingredients	Number of commenters addressed (*n* 1125)	Exemplar comments shared with the FDA
Dietary components recommended to include		
Plant-based whole foods (fruits, vegetables, nuts, seeds and grains)	291	*Healthy tends to be fruit, vegetable, nuts, and within reason (portion matters) seafood and meats. –* [Individual consumer, North Carolina] *If ‘Healthy’ is Used, Focus on Plant Foods… Singling out plant-based foods is not an attempt to say that these are the only foods that should be eaten, but simply to recognize that most Americans would benefit from a constant reminder to eat more plant- based whole foods, whatever other choices they are making in their diets. –* [International organization, location not specified]
Dairy and animal-source foods	170	*Lean meat gives healthy fats and protein to the body. –* [Individual consumer, location not specified] *Dairy’s nutrient package includes nutrients under-consumed by most Americans—calcium, vitamin D and potassium—as well as high-quality protein, phosphorus, magnesium, zinc, vitamin B-12, vitamin A, riboflavin and choline. –* [Food Association, location not specified] *Meat, dairy, eggs and seafood are huge contributors of ‘bad’ fat and cholesterol, the intake of which should be limited as much as possible in a healthy diet… –* [Individual consumer, location not specified]
Oils	79	*We need fat in our diets, preferably from unsaturated fats like the ones found in nuts, seeds, fatty fish, avocados, and vegetable oils. These are actually considered to be healthy since they are so beneficial to our diets. –* [Academia, location not specified] *With some of the more recent studies, the definition of fats need to be redefined to include good, quality, healthy fats (nuts, avocado, olive)… –* [Individual consumer, location not specified] *The public has a wrong impression on fats, they are healthy as long as they are the right kind of fats - coconut oil, olive oil, real butter, nuts, and eggs. –* [Individual consumer, location not specified]
Dietary components recommended to limit		
Added sugars, sodium and saturated and *trans* fats	493	*Healthy foods do not have artificial colors, artificial flavors, MSG, GMO ingredients, preservatives, hydrolyzed oils, high fructose corn syrup, artificial sweeteners, like sucralose [sic]. –* [Individual consumer, location not specified] *‘Healthy’ should be low in fat, sodium, cholesterol, and/or sugar. It should be made with minimally processed ingredients. –* [Individual consumer, Florida] *Food with saturated fats are unhealthy*. *–* [Individual consumer, Texas] *[Z]ero trans fats are healthy (not the current 0·5 g that is now allowed as being zero)*. *–* [Individual consumer, location not specified]

Reflecting the intent of the request for comment to gather feedback to inform labelling rules, almost half of commenters discussed food processing (*n* 542, data not shown), largely emphasising the healthfulness of unprocessed or minimally processed ‘whole’ foods. For example, one individual consumer (location not specified) noted that healthy food is ‘natural, made up of ingredients that came from nature and are as raw and unaltered as possible.’ Another individual consumer (location not specified) shared ‘Any food products that are processed and packaged in any way should be disqualified… from using the “healthy” label.’

Over one-third of commenters (*n* 493) shared that healthy foods contain limited or no added sugars, sodium, saturated fats and/or *trans* fats. Some commenters (*n* 297, data not shown) also were concerned about the inclusion of food additives, including preservatives, sweeteners and dyes during processing. These comments were more common among individual consumers than respondents from other reporting categories. A few commenters (*n* 43, data not shown) mentioned the importance of understanding what constitutes a serving and selecting an appropriate amount based on dietary needs to reduce overconsumption. For example, one individual consumer (location not specified) wrote, ‘[S]erving sizes need to be reevaluated, so that people get their nutrition facts based on a realistic portion size.’

### Consideration of sustainability in comments

Fewer than 3 % of submissions (*n* 30) included the terms ‘sustainable,’ ‘sustainably’ or ‘sustainability,’ but approximately one-third of commenters referenced one or more dimensions of sustainability. For example, although they did not mention sustainability, one individual consumer (location not specified) articulated how their understanding of ‘healthy’ extends far beyond nutrition content: ‘Healthy food means much more than what food does for you after you consume it. Truly healthy food is the finished product of a healthy process. This means the health of the producers, processors, distributors, retailers and consumers is added to the definition. A food may contain high amounts of vitamins, fibre or whole grain, but if the process in which it got from farm to fork excludes the health of the workers and the planet, can it be healthy in the true sense of the word?’ We present the frequency with which commenters addressed issues aligned with each dimension of sustainability in Table 2 and describe the nature of the comments below. Of the three dimensions of sustainability, aspects of environmental sustainability were referenced the most frequently.

**Table 2:** Public submissions to FDA-2016-D-2335 that address environmental, social and economic dimensions of sustainability

Dimension of sustainability	All commenters (*n* 1125)		Individual commenters (*n* 766)	
	*n*	%	*n*	%
Any	374	33·2 %	325	42·4 %
Environmental sustainability	252	22·4 %	225	29·4 %
Social sustainability	187	16·6 %	126	16·4 %
Economic sustainability	7	0·6 %	4	0·5 %

#### Environmental sustainability

About one in five commenters (*n* 252), most commonly individual consumers, described considerations aligned with the environmental dimension of sustainability. These commenters primarily shared concerns with conventional farming. Their concerns centered on agrochemical use and GM organisms (GMO) and often took the form of appeals for organic agriculture. Commenters advocated for an end to the use of pesticides, herbicides, antibiotics, hormones, other ‘chemicals’ and GMO in food production and felt strongly that any products that were not organic and GMO-free should not bear a ‘healthy’ label.

Commenters expressed worries about contamination of the food supply and compromised food safety. One concern related to agrochemical ‘residues’ making their way into people’s diets, as articulated by an individual consumer (location not specified): ‘The level of pesticide and chemical residues present in non-organic produce and processed foods is a problem. We don’t have enough scientific information yet on the long term consequences of their presence let alone how they will interact with each other – but it can’t be good. Unnatural chemicals floating into your body… also the impact on ground water and our soil is not fully understood or given enough consideration.’ Another concern pertained to the inclusion of GM ingredients in the food supply and lack of labelling as such. For example, a health professional (location not specified) wrote, ‘The word ‘Healthy’ or ‘Natural’ should only be allowed to be used when it is healthy or natural, or in other words only ORGANIC foods such as organic grains and produce, wild caught fish, grass fed meat or raw dairy/produce etc… Anything else sprayed or treated or especially GM is not healthy nor natural and does not qualify as food.’ At the time of the request for comment, no national standard existed for disclosing foods that are GM or may contain GM material. Mandatory compliance for the National Bioengineered Food Disclosure Standard (established in December 2018) began 1 January 2022^(42)^.

Other comments raised by a smaller number of commenters considered (1) whether the living conditions, diets and well-being of livestock should be considered as part of the definition of ‘healthy’ and (2) if knowing a food’s origin is pertinent to determining its healthfulness. Although, as noted above, numerous submissions emphasised the benefits of eating a diet rich in plant-based foods and a few recommended reducing or eliminating meat intake; only two commenters explicitly mentioned links between dietary pattern and environmental sustainability.

#### Social sustainability

About one in six comments (*n* 187) addressed themes relevant to social sustainability. Most of these focused on the power of the food industry, especially producers of processed and packaged foods, and the ways in which this may affect the healthfulness of the food supply. Some commenters raised concerns about the influence of the food industry within government policymaking and rulemaking and/or stated that they believe there is a conflict between food industry profits and public health goals. An illustrative quote from an individual consumer in Colorado was, ‘It’s time the FDA listened to the nutritionists who work on behalf of the public instead of agri-giants, chemical companies and food processors.’ Another stated, ‘It is the job of the government to protect its people and their rights. How can a nation be expected to make wise choices when it comes to eating if they are falsely informed or if the information is simply disregarded or stretched for the benefit of large industries and companies?’ A small number of submitters shared an alternative opinion, expressing the belief that the government should not regulate the food supply, emphasising the benefits of individual judgement about what is healthy.

Other submissions relevant to social sustainability addressed the need to protect and promote the well-being of people involved in the food system from primary production through final consumption. These comments came primarily from individual consumers and academic submitters. Comments on the well-being of food and farm workers stated the importance of decent livelihoods and safe working conditions, as well as support for food systems that bring value to communities. For example, one individual consumer in Connecticut shared, ‘Healthy food is produced sustainably, using methods that neither deplete resources nor exploit farmers and farm workers.’ Comments related to consumer well-being considered food as a basic human right. These primarily emphasised the need to ensure all people have access to accurate nutrition information and affordable, nutritious food that meets their preferences. For example, an individual consumer from Maryland wrote, ‘Healthy food means that the individual is receiving a sufficient level of energy and a full array of macro- and micronutrients needed to thrive physically. At the same time, the individual is eating foods that align with their culture, preferences, values and means.’

#### Economic sustainability

Only seven comments raised issues related to economic sustainability. These addressed two topics: how local food systems can contribute to ‘a strong local economy’ and how labelling rules may affect the bottom line of food businesses.

## Discussion

Similar to the definition of a ‘healthy eating pattern’ as outlined in the DGA^(40)^, this study found individuals that submitted comments to the FDA widely recognised vegetables, fruits, whole grains and other unprocessed or minimally processed ‘whole’ foods as ‘healthy’ and identified added sugars, sodium and saturated and *trans* fats as ingredients to limit. Notably, one-third of commenters addressed one or more dimensions of sustainability *beyond* nutrition when defining ‘healthy,’ even when the term ‘sustainability’ was not specifically used.

Public comments that did address sustainability primarily alluded to environmental issues. Among these, concerns about food safety, specifically contamination of the food supply by agricultural inputs, GMO or ingredients introduced during the processing of packaged foods were mentioned most frequently. Commenters rarely mentioned other environmental aspects of food system sustainability, such as food waste, long-distance distribution networks and/or single-use packaging waste. National attention on recent federal proposals and rulemaking on bioengineered/GMO and ‘natural’ labelling could be one reason for commenters’ focus on organic production and unprocessed or minimally-processed foods^(43)^. Non-environmental dimensions of sustainability were less frequently mentioned, suggesting that the prioritisation of environmental sustainability in research and advocacy on food systems^(44)^ has contributed to greater public awareness of this dimension. Of the comments that did raise non-environmental dimensions of sustainability, comments noted the potential influence of larger agri-food businesses in the policy process. The fact that social and economic issues were less commonly mentioned by commenters suggests that these commenters consider a food’s environmental impacts more relevant to its healthfulness than its social and economic impacts and/or that more work is needed to understand and illuminate all dimensions of food system sustainability, especially those related to economic resilience and social well-being. Prior research indicates that sustainability considerations – especially environmental considerations – ‘largely left out’ of national dietary guidelines, including the DGA^(12,14)^.

The findings of this study as well as public comments submitted in response to the 2015 DGAC scientific report^(22)^ suggest that some members of the public believe that policy makers should consider sustainability dimensions when developing nutrition policies and regulation designed to promote healthier food choices, including the DGA. To date, a common proposed solution to the challenges of unhealthy diets and diet-related chronic disease has been individual-level behaviour change through education and guidelines, including food labelling efforts. While labelling may empower some consumers^(45)^, it also has the potential to reinforce socio-economic inequities in purchasing and consuming behaviours, as a myriad of social, economic and system factors can influence food choice and dietary patterns. There is accumulating evidence that interventions that require less effort on the part of consumers may be more effective and equitable^(46)^. With consideration of current and cumulative evidence, policy measures designed to support ease of healthy dietary purchase and consumption patterns aligned with achieving one or more dimensions of food system sustainability could be considered. Since the DGA underpins many federal food, nutrition and health policies and programs in the USA that is one among the clear opportunities to consider.

The dataset used in this study may limit the external validity of the findings; the portion of the population that was aware of this docket and motivated to submit a comment is unlikely to be representative of the US population. Documented barriers to participation in federal rulemaking by ordinary citizens include lack of awareness that rulemakings of interest are going on, difficulty reviewing rulemaking materials and limited understanding of how to participate effectively^(47)^. Additionally, poor/limited internet access among some population subgroups, including socioeconomically under-resourced and geographically isolated populations, could hinder participation^(48)^. However, commenters to this proposed rule came from across the country and expressed a broad range of views, suggesting that the sample captured some of the diversity of the US population. A distinctive aspect of this sample is that it was comprised primarily of individual consumers and contained few form letters. With few exceptions, each submission was unique. Prior research has found that federal agencies place little value on form letters, but appreciate original, substantive comments^(49,50)^. In fact, government guidance specifies that ‘one well supported comment is often more influential than a thousand form letters’^(51)^. This suggests that unique submissions like those reviewed for this study will carry greater weight with agency rule makers. Future research should explore how views expressed by individuals who submitted comments to the Federal Register differ from those of individuals who did not submit a comment and investigate the source and quality of evidence used to support claims made by commenters.

Internal validity of perceptions regarding sustainability may have been limited by the focus of this request for comment. In particular, some submissions may have overlooked issues related to sustainability because participation may have been prompted by a citizen petition, which focused on the nutrient content claim ‘healthy,’ not overall diet quality^(37)^. However, we believe that the questions asked by FDA were sufficiently broad to welcome diverse submissions on the topic, evidenced by our finding that one in three comments considered at least one dimension of sustainability.

This paper adds to recent evidence suggesting that public comments can provide useful data for policy-relevant public health nutrition research^(52–57)^. Such data provide one potential pathway to understand public perceptions and may be useful to complement other methods such as survey research and social media data mining. Triangulation across multiple types of data may help overcome each method’s limitations and present a more complete view of public opinion on if and how health and food system sustainability are connected. Several strengths of the present study are worth noting. First, this research is extremely timely. The FDA has not yet finalised the new definition of the term ‘healthy’ and is actively developing a symbol that the food industry can voluntarily use to label food products that meet the updated definition of ‘healthy’^(39)^. Second, we applied a rigorous approach to coding and analysis that involved training all coders in person, establishing consistent application of the codebook prior to full coding and collaborative reviews and discussion of all coding and code memos. Third, unlike some previous public health nutrition studies using Federal Register data^(53,55)^, we analysed all submitted comments and thus were able to observe the full breadth of submissions and examine a large dataset. Finally, we adopted a team-based process, which is both more inclusive and supports more comprehensive interpretation than if the final analysis was conducted by only one or two authors.

### Conclusions

This research sheds light on salient population-level nutrition and food sustainability perspectives and considerations. Specifically, those who participated in this invitation for public comment generally defined ‘healthy’ foods and ingredients in a manner similar to how a healthy eating pattern is defined by the DGA, suggesting that the FDA’s proposal to better align labelling regulation for ‘healthy’ with the DGA reflects public opinion. However, of note, one in three individuals who shared their views with the FDA also consider ‘healthy’ foods to embody certain attributes of sustainability, particularly environmental aspects, and consider these factors in their own purchasing and eating behaviours. Thus, further discussion and policy consideration is warranted, as it is not currently represented in how the DGA and FDA currently conceptualise ‘healthy’ food.

## Supporting information

Belarmino et al. supplementary material Belarmino et al. supplementary material

## Supplementary material

For supplementary material accompanying this paper visit https://doi.org/10.1017/S1368980024000636
