# Supplementary material for: Consideration of nutrition and sustainability in public definitions of ‘healthy’ food: an analysis of submissions to the US FDA
Source: Public Health Nutr. 2024 Apr 4;27(1):e119. doi: 10.1017/S1368980024000636 (PMC11036447; doi:10.1017/S1368980024000636)
Supplement: Belarmino et al. supplementary material [file S1368980024000636sup001.docx]

Supplementary File 1.

Descriptive coding frames relevant to nutrition and sustainability that were applied to public comments submitted to the U.S. Food and Drug Administration in 2016 and 2017 in response to a request for public comment on use of the term “healthy” on food labels

**Dietary factors in the 2015-2020 Dietary Guidelines for Americans**

| Code name | Short description | When to use (inclusion criteria) | When not to use (exclusion criteria) | Examples of data that represent the code |
| --- | --- | --- | --- | --- |
| **Included in a “healthy dietary pattern” as defined in the DGA** | | | | |
| 1. Plant-based foods | | | | |
| a. CARBS_VEGGIE | Vegetable consumption | Use when individuals comment on vegetables |  | “Healthy is mostly vegan, organic diet full of dark leafy greens, green teas, and plant protein.” |
| b. CARBS_FRUIT | Fruit consumption | Use when individuals comment on fruit |  | “Whole grains, nuts, and real fruit and no food dyes should be considered healthy.”  “Concentrated fruit sugars (not eaten as whole fruit) are also unhealthy.” |
| c. CARBS_GRAINS | Grain consumption | Use when individuals comment on grains. Use for all mentions of whole and refined grains |  | “Studies suggest whole-grain foods are beneficial to cardiovascular health and can help prevent chronic diseases.”  “Whole grains, nuts, and real fruit and no food dyes should be considered healthy.” |
| d. PROTEIN_PLANT_DIET | Plant-based diet | Use when individuals comment on consumption of plant-based proteins (e.g., nuts, seeds, legumes). Also use for comments on plant-based diets when the comment specifies the merits/demerits of whole plant-based foods (without specifying the food). |  | “Healthy is mostly vegan, organic diet full of dark leafy greens, green teas, and plant protein.”  “Whole grains, nuts, and real fruit and no food dyes should be considered healthy.”  “Soy is unhealthy for men, since it has components that stimulate estrogen.” |
| 2. Animal-source foods | | | | |
| a. PROTEIN_DAIRY | Dairy products | Use when individuals mention any type of dairy consumption |  | “I find it alarming and misleading that meat, milk and dairy products are pushed upon individuals as a result of lobbying and corruption.”  “Additionally, added sugars should be taken into consideration, because many individuals think the yogurt they eat is "healthy" but don't take into consideration how much added sugar is in their diet and how it contributes to excess calories.” |
| b. PROTEIN_MEAT | Meat, seafood, eggs | Use when individuals comment on consumption of meat, seafood, or eggs. |  | “Healthy is the elimination of red meats”  “I find it alarming and misleading that meat, milk and dairy products are pushed upon individuals as a result of lobbying and corruption.”  “Some meats are healthier than others. Fish is a bit healthier than chicken, which is much healthier than lamb or beef, which are healthier than pork, according to my own analysis of mortality and consumption rates.“ |
| 3. Oils | | | | |
| a. FAT_OILS | Oils | Use when individuals comment on consumption of oils | Do not use for hydrogenated oils (code at FAT_SAT_TRANS) | “Ingredients identified as high risk for disease should be excluded, this would exclude all hydrogenated vegetable oils, partially-hydrogenated vegetable oils, and brominated oils.” |
| **Limited in a “healthy eating pattern” as defined in the DGA** | | | | |
| 4. SUGAR_ADDED | Added sugar | Use when individuals comment on “added sugars.” Also use for mentions of high fructose corn syrup. When comments mention artificial sweeteners, code here and at PROCESSED_ARTIFICIAL |  | “Additionally, added sugars should be taken into consideration, because many individuals think the yogurt they eat is "healthy" but don't take into consideration how much added sugar is in their diet and how it contributes to excess calories.” |
| 5. FAT_SAT_TRANS | Saturated or trans fat | Use when individuals comment on consumption of saturated or trans fats. Also use for mentions of hydrogenated fats or oils. |  | “No trans fats” |
| 6. SODIUM | Sodium content in foods or diets | Use when individuals comment on sodium or salt in foods or diets | . | “Therefore, sodium should be included in the definition of healthy since Americans typically consume way too much sodium in their diet.”  “Low salt”  “Organic in nature with less additives, including low sugar, fat and salt.” |
| **Other relevant codes** | | | | |
| 7. PROCESSED | Level of processing | Use when individuals comment on levels of processing, i.e., minimal, none at all. Also use for mentions of “fresh” or “whole” food. Use this code when fortification is mentioned. | Do not use for comments on food additives or “chemicals” in food (code as PROCESSED_ARTIFICIAL). Do not use for mentions of refined grains (code as CARBS_GRAINS) | “Nuts, seeds, eggs, etc. can be healthy assuming minimal processing.”  “It means all natural (preferably organic), with a short list of ingredients that have gone through as little processing as possible.” |
| a. PROCESSED_ARTIFICIAL | Additives to food | Use when individuals comment on substances added to food to enhance taste or appearance or act as a preservative. Code references to “chemicals” in food here. When artificial sweeteners are mentioned, code here and at SUGAR_ADDED. | Do not use for high fructose corn syrup (code as SUGAR_ADDED). | “Healthy food should not contain artificial colors, flavors, ingredients or cellulose/fillers.”  “High natural fiber content (not artificially added fiber).“  “Organic in nature with less additives, including low sugar, fat and salt.”  “A “HEALTHY” food or drink is as follows: No preservatives unless it’s organic using plant and packaging technology NOT CHEMICALS!” |
| 8. SERVING_SIZE | Serving size of foods | Use when individuals comment on serving size. |  | “In terms of the profile, the serving size should be representative of what a human would eat in a serving.“ |

**Food system sustainability**

| Code name | Short description | When to use (inclusion criteria) | When not to use (exclusion criteria) | Examples of data that represent the code |
| --- | --- | --- | --- | --- |
| 1. ENV INTEGRITY | Environmental integrity – preservation or degradation of the environment | Use for comments that address environmental integrity, but are not captured by the available child codes. |  | “Healthy food enriches our environment; it doesn't destroy it.”  “A food may contain high amounts of vitamins, fiber, or whole grain, but if the process in which it got from farm to fork excludes the health of the workers and the planet, can it be healthy in the true sense of the word?” |
| a. NATURAL RESOURCES | Natural resources – the integrity and preservation of natural resources | Use for comments that address the integrity and preservation of natural resources, but are not captured by the available child codes. |  | “Healthy food is produced sustainably, using methods that neither deplete resources or exploit farmers and farm workers.” |
| i. ENV CONTAMINANTS | Environmental contaminants and pollutants | Use for comments about environmental contaminants and/or pollutants used in the food system. These include nutrients (i.e., nitrogen and phosphorus), pesticides (including mentions of organic foods/production), pharmaceuticals (e.g., antibiotics, hormones), odors, and fine particulate matter.  Also use this code when environmental “chemicals” are mentioned. | Do not use for references to chemicals used to preserve food.  With the exception of odors and particulate matter, do not use for references to air pollution (code as CLIMATE CHANGE). | “Chemicals used to produce more or better food from an acre are necessary. However those chemicals should not harm the environment.” |
| ii. SOIL AND WATER | Depletion and replenishment of arable land and water | Use for comments about factors influencing the availability and quality of soil and water and the balance of loss and regeneration of these resources. This may include references to changes due to human decisions (e.g. excessive soil erosion due to improper tillage practices) and factors outside human control (e.g. floods and droughts). Comments on composting and recycling water should be coded here. | Do not use for mentions of pesticides and fertilizers. Do not use for any comments about soil quality being diminished by pesticides/fertilizers (code as ENV CONTAMINANTS). | “Healthy food is also healthy for the land, water, and community where it is produced (what is sustainable in one climate/community, isn't necessarily sustainable in another).”  “It is harvested through sustainable practices that threaten neither the soil nor the water nor the air.” |
| iii. CLIMATE CHANGE | Food system contribution to climate change | Use for references to food system activities, including producing food, transporting it, and storing wasted food in landfills, produce greenhouse gas emissions that contribute to climate change. Also use to capture references of the ways in which climate change impacts agriculture. |  | “Food that is locally-sourced and does not contribute significantly to carbon emissions (note: the true cost of food should be included on its label, a kind of "carbon calorie" that would reveal how great the carbon emissions were to get that piece of food to your location).” |
| b. BIODIVERSITY | Biodiversity – the diversity of ecosystems, of species in these ecosystems and of the genome within these species | Use for references to the impact of food systems on biodiversity, including the disruption of population and community dynamics among species within ecosystems. Also use this code for all references to genetic modification, as a major argument against genetic modification is that it adversely impacts biodiversity. |  | “The term "healthy" should only be used on whole organic foods that are pesticide and GMO free, with no preservatives, dyes or other additives. Otherwise the term "healthy" is a lie.”  “I don't think it's critical to take "organic" or "non-GMO" into consideration, as manufactureres add this information on their labels voluntarily.” |
| c. MATERIALS AND ENERGY | Materials and energy used in the food system, particularly with regard to input efficiency | Use for comments that address materials and energy, but are not captured by the available child codes. |  | “Let's encourage people to eat something besides an american "Cheese" slice that comes in an individual plastic wrapper (which is a whole different issue!)” [NOTE: this is the only comment coded at MATERIALS AND ENERGY.] |
| i. FOOD WASTE | Food waste | Use for comments regarding food losses during production, post-harvest, and processing, as well as food waste that occurs at marketing and consumer levels. |  | “Since Healthy Food is more valuable than its counterparts and contains no preservatives or additives to make it last. We must also take steps to prevent waste to create an efficient and healthy food system. An increased reliance on local agriculture would also help to reduce waste as local farmers could adapt their food production to address consumer needs.” |
| d. ANIMAL WELFARE | Animal welfare – the physical and psychological well-being of animals | Use for comments regarding livestock production under conditions appropriate or inappropriate for animal welfare and health. This includes keeping animals free from hunger, thirst, injury, disease, discomfort, pain, fear, or distress, and ensuring that animals are free to express normal behavior.  Use for comments about the overall ethics of livestock production. |  | “Healthy animal foods are NOT the animal foods produced by huge industrial farms. Healthy animal products should come from cows, chickens, etc who are living a natural life - not stuffed into warehouses or tiny, overcrowded coops.” |
| e. FOOD ORIGIN | Source or origin of food or food products | Use for references to where food came from or its traceability as a factor related to healthfulness or sustainability.  Use for references to local foods where the emphasis is not on impacts for the local economy or social welfare.  Use for references to seasonality. | Do not use for references that focus on local economic or social welfare impacts (code as LOCAL ECONOMY or at one of the child codes of SOCIAL WEFARE).  Do not use for references to “food miles” or fossil fuels used in long distance food transport (code at CLIMATE CHANGE). | “Healthy food is food that you can point to and say, "This is where it came from and I know who farmed or caught it."” |
| 2. ECON RESILIENCE | Economic resilience –ability to cover costs (including negative externalities), generate a positive cash flow, and adequately remunerate workers and shareholders, as well as have savings or assets to cope with changes and shocks | Do not use. |  |  |
| a. GEN ECON RESILIENCE | General economic resilience | Use for comments regarding economic investment, profitability, and vulnerability to withstand and be adapted to environmental, social, and economic shocks. |  | “Costs to industry could be expected to heighten during the transformation as food manufacturers will have to adapt to the change in consumers and will have to alter their nutritional values, ingredients, and reflective labels in order to remain competitive. Ultimately, this change is a step in the right direction of not only meeting the nutritional goals of individuals, but also the health goals of the country.” |
| b. LOCAL ECONOMY | Local economy | Use for references to the impact of the food system to local economic development, including impacts through employment, payment of taxes, and procurement through local suppliers. | Do not use for comments on the livelihoods/incomes of food producers and employees in the food system (code as ECON LIVELIHOOD). | “Healthy food means eating in a way that improves our personal, community, and environmental health over time. Healthy food is fresh, local, seasonal, and as close to the earth as we can get it. Healthy food contributes to a strong body and mind, a strong local economy, and a strong and resilient environment.” |
| 3. SOCIAL WELFARE | Social welfare – the well-being of food producers and employees in the food system | Use for comments on social welfare that are not captured by child codes. |  | [This code was not used.] |
| a. GOVERNANCE | Governance – how the food system is controlled pre-consumer; the process of making and implementing decisions | Use for comments related to the power of stakeholders in the food system and accountability within the food system that are not captured by the child codes. | Do not use this code for references to misleading labeling.  Do not apply this code for references to consumer decision-making. | “I would like to voice my support for proposed rule-making by the FDA to regulate the use of terms, such as "healthy", on food product packaging.”  “Healthy is a broad term describing an accumulation of lifestyle choices. Designating any one food as healthy is capricious and irresponsible. Please consider prohibiting use of this term for foods.” |
| i. INDUSTRY ETHICS/INFLUENCE | Influence of the food industry in the food system | Use for comments that refer to the power and responsibility that the food industry has within the food system. Also, use for comments on food industry ethics.  Comments about the role of lobbyists and/or other representatives of the food industry in interacting with the government should also be coded at GOVERNMENT ETHICS/INFLUENCE. |  | “It is better if no health claims be allowed on any products than to allow food manufacturers to find a way to game the system.”  “Butt out. Let the label represent the manufacturer's opinion of whether the food is healthy or not, not the opinion of some panel of bureaucrats wasting my money. Lay off the whole division in charge of this effort and cut my taxes.” |
| ii. GOVERNMENT ETHICS/INFLUENCE | Influence of the FDA/government in the food system | Use for comments that refer to the power and responsibility of the FDA/government in the food system.  Also, use for comments on FDA/government ethics within the food system. Comments may relate to interaction with lobbyists and/or other representatives of the food industry. These comments should also be coded at INDUSTRY ETHICS/INFLUENCE.  Also use this code when lack of trust in government or dislike of big government is mentioned. |  | “Good for the FDA for calling out KIND bars. Tasty but not healthy. You're on the right track. You can't make American eaters smarter but you can hold manufacturers to a high standard of truth.”  “It's incredibly important for the FDA to support Americans getting the information in order to live a healthy lifestyle…I highly support redefining "healthy" food labels, and strongly encourage the FDA to move forward for the good of the American people.”  “Butt out. Let the label represent the manufacturer's opinion of whether the food is healthy or not, not the opinion of some panel of bureaucrats wasting my money. Lay off the whole division in charge of this effort and cut my taxes.” |
| iii. EXPERT INFLUENCE | Role of nutrition/science experts in determining what is healthy. | Use for comments that refer the role that nutrition experts/scientists should have in guiding decisions in the food system. |  | “I don't think the food industry or public's idea of what warrants a "healthy" label on food packaging is relevant. The FDA should be soliciting the opinion of nutrition scientists who have empirical evidence to back up their claims.” |
| b. ECON LIVELIHOOD | Economic livelihood – access to a safe, decent standard of living for health and well-being for food producers and employees in the food system | Use for references to producers’ and employees’ quality of life, access to professional development opportunities, and access to means of production, including equipment, capital, and knowledge. |  | “Healthy is fair to producers. Food that does not harm the people who grow it. Producers are compensated fairly for their time and effort so as to encourage agriculture that can be clean.” |
| c. ETHICAL FOOD SYSTEM | Ethical food system – respect and rights for food producers, their employees, and consumers | Use for references to fair trading practices, labor rights, equity (including gender equity, non-discrimination, and support to vulnerable people), and workplace health and safety.  Use for comments regarding the affordability of various foods (from the consumer’s perspective). |  | “Healthy food is a human right. Healthy food makes your body feel good.”  “If you want the population to create good dietary patterns, make fresh produce more available and affordable. People know they're supposed to eat mostly fruits and vegetables but not everyone has equal access or the income to afford to feed their whole family a whole food diet.” |
| d. CULTURAL DIVERSITY | Cultural diversity – the existence and protection of a variety of cultural and ethnic groups, practices, and foods within the food system | Use for references to the protection and recognition of intellectual property rights related to traditional and cultural knowledge and references to food sovereignty, i.e. the right to choice and ownership of production means, such as the preservation and use of traditional, heirloom, and locally adapted varieties and breeds. |  | “Healthy food means that the individual is receiving a sufficient level of energy and a full array of macro- and micronutrients needed to thrive physically. At the same time, the individual is eating foods that align with their culture, preferences, values, and means.” |
